# Multifamily groups for patients with schizophrenia: an exploratory randomised controlled trial in Bosnia and Herzegovina

**DOI:** 10.1007/s00127-022-02227-9

**Published:** 2022-02-12

**Authors:** M. Muhić, S. Janković, H. Sikira, S. Slatina Murga, M. McGrath, C. Fung, S. Priebe, A. Džubur Kulenović

**Affiliations:** 1 Department of Psychiatry, University of Sarajevo, Sarajevo, Bosnia and Herzegovina; 2 Faculty of Medical Sciences, University of Kragujevac, Kragujevac, Serbia; 3 Unit for Social and Community Psychiatry, WHO Collaborating Centre for Mental Health Services Development, Queen Mary University of London, London, UK

**Keywords:** Multifamily groups, Resource-oriented interventions, Psychoeducation, Trialogue, Schizophrenia

## Abstract

**Background:**

Schizophrenia has a significant and lasting impact on the quality of life of patients and their families and is a leading cause of disability globally. Family interventions can be beneficial and may be particularly appropriate in settings with limited resources. We conducted an exploratory trial testing the effectiveness of a multifamily group intervention, which draws on the traditions of psychoeducation and trialogue, for improving the quality of life of patients with schizophrenia in Bosnia and Herzegovina.

**Methods:**

We conducted an exploratory, randomised controlled trial with patients with schizophrenia attending an outpatient clinic in Sarajevo. Our primary outcome was improved quality of life at 6-month follow-up. Secondary outcomes included objective social outcomes, psychiatric symptoms and psychiatric hospitalisation measured at 6 and 12 months. Experiences of participants were assessed in post-intervention interviews.

**Results:**

72 patients were randomly assigned to either one of six multifamily groups or treatment as usual. Follow-up assessments were completed with 53 patients (74%) at 6 months and 55 patients (76%) at 12 months. The intervention significantly improved quality of life at 6 months (Cohen’s *d* = 0.78, *F* = 6.37, *p* = 0.016) and 12 months (*d* = 1.08, *F* = 17.92, *p* < 0.001), compared with treatment as usual. Re-hospitalisation rates at 6 months and symptom levels also improved significantly whilst changes in other secondary outcomes failed to reach statistical significance.

**Conclusion:**

These findings suggest multifamily groups can be effective for improving the quality of life of patients with schizophrenia in Bosnia and Herzegovina. Further research is required to assess how multifamily groups may be scaled up in similar settings with limited resources.

## Introduction

An estimated 21 million people are living with schizophrenia globally [1]. Schizophrenia and related disorders lead to higher mortality, are a leading cause for disability [2, 3], and can have a substantial and lasting impact on the quality of life of patients and their families. Many people living with schizophrenia experience poorer social outcomes, such as unemployment, insecure housing, poverty, social stigma and discrimination, and strained relationships with families and caregivers [4–6]. The burden of disease attributable to schizophrenia in low- and middle-income countries is around four times that experienced in high-income countries [1], and effective low-cost interventions are required to improve outcomes in these settings.

Family involvement interventions, in combination with pharmacotherapy, are a recommended treatment for people with schizophrenia and related psychotic disorders [7, 8], and empowering patients and family members as active participants in the design and delivery of mental health services has been highlighted as a global priority [9]. Utilising personal and social resources within patients and their families and the sharing of experiential knowledge are potential mechanisms for improving quality of life in people with schizophrenia [10]. Interventions that promote greater family involvement have been shown to reduce relapse and hospitalisations, and increase medication adherence in people with schizophrenia [11, 12]. Further, these interventions appear to promote greater patient satisfaction with treatment [13, 14] and improve social functioning [11].

Nevertheless, in many low- and middle-income countries, standard care for people living with schizophrenia consists mainly of pharmacotherapy. Persistent underinvestment in mental health care, limited mental health workforce and contextual barriers to service availability and accessibility have contributed to a large mental health treatment gap globally [15, 16]. Lack of treatment can be associated with poorer long-term clinical outcomes and increased disability [17]. Using family interventions in such contexts requires a low-cost and easy to implement model.

Typical for many low-resource settings is the situation in Bosnia and Herzegovina. The quality of mental health care deteriorated substantially following the Bosnian War, due to the destruction of large health institutions, reductions in the number of qualified mental health professionals, and widespread damage to social networks, families, and other support systems [18]. A scarcity of mental health professional limits the delivery of services, with 34 mental health workers per 100,000 population, compared with 156 per 100,000 in Western Europe [19]. Stigma, segregation and social isolation represent fundamental barriers to the treatment and recovery of people with mental disorders [20]. There are no resources to establish expensive specialised services, and effective low-cost family interventions may be a feasible approach to improve care.

We developed and tested a brief, multifamily group intervention for patients with schizophrenia and related disorders in Bosnia and Herzegovina. Multifamily groups bring together patients with schizophrenia, their family and friends, and mental health professionals. Our model draws on the traditions of trialogue and psychosis seminars, where learning occurs through the sharing of experiences [21–23]. The groups also mobilise mutual support and provide some elements of psychoeducation. For a total period of 6 months, the groups come together once per month and discuss topics selected by participants based on their priorities and interests.

We conducted an exploratory randomised controlled trial on the effectiveness of this multifamily group intervention for patients with schizophrenia in Bosnia and Herzegovina.

## Methods

### Study design and participants

Between March 2018 and August 2020, we conducted a parallel-group, randomised controlled trial of multifamily groups for patients with schizophrenia in Sarajevo, Bosnia and Herzegovina.

Clinicians at the Clinical Centre at the University of Sarajevo screened their medical records for patients with schizophrenia treated on an outpatient basis. Patients were eligible if they were 18 years or older, had a primary diagnosis of schizophrenia or non-affective psychosis (ICD-10: F20–29), were currently outpatients (i.e. not hospitalised) and were not participating in another research study. Eligible patients were approached by trained researchers and, if they provided written informed consent, were screened for subjective quality of life. To exclude patients with a very high subjective quality of life at the beginning, only patients with a mean Manchester Short Assessment of Quality Life score of ≤ 5 were included. Patients meeting these criteria who agreed to participate were asked to nominate one or two family members or friends with whom they would like to attend sessions.

The trial was prospectively registered with the ISRCTN Registry (ISRCTN13347355) and ethical approval obtained from the University of Sarajevo and Queen Mary University of London. Further details are outlined in a published protocol [24].

### Procedure

Patients with their family members and friends were randomly allocated to one of six multifamily groups—in addition to treatment as usual—or treatment as usual alone. Randomisation was conducted by an independent researcher using sequential, computer-generated random numbers and allocation information was provided to an unblinded research coordinator at the site.

In the intervention group patients attended multifamily group meetings, which consisted of 1–2 clinicians, 5–6 patients and the 1–2 family members or friends that each patient had selected to participate in the intervention. The intervention aimed to utilise resources in patients and their families by encouraging mutual learning and the exchange of experiences. Groups met once a month for 6 months at the Psychiatric Clinic of the University of Sarajevo and sessions were scheduled for 2 h including a 10-min break. Meetings were usually chaired by a mental health professional; however, some groups decided to nominate a patient or family member to chair the meeting or to rotate the role. Other than some basic rules to encourage mutual respect, sessions were organised flexibly to accommodate the priorities of participants. At each meeting, a mix of pre-defined and participant-generated topics was chosen for discussion. Patients in both intervention and control arms continued to receive treatment as usual.

Patient baseline assessments were completed by the study coordinator and 6- and 12-month post-enrolment follow-up assessments by blinded researchers. As the impact of the intervention on the patients is the focus on this paper, only outcomes in the patients are reported here. In each assessment period, up to four attempts were made to conduct follow-up assessments. Data were collected using a standardised, paper case report form developed for each group (patients, family members/friends, clinicians) and were later entered into a REDCap database for secure storage and analysis.

Qualitative interviews were conducted in Bosnian with patients at 6 months. The research team developed an interview topic guide to explore the experiences of the intervention, barriers and facilitators, proposed adaptations, and practical issues around implementation. Each interview was audio recorded and lasted 45–60 min.

### Outcomes

The primary outcome was subjective quality of life at 6 months measured using MANSA (Manchester Short Assessment of Quality of Life), an instrument which has been widely used in mental health research. A mean score is calculated from the 12 items measuring satisfaction with life domains, which are rated by the patient from 1 (‘couldn’t be worse’) and 7 (‘couldn’t be better’) [25].

Secondary outcomes were psychiatric symptoms, objective social outcomes and mental health service utilisation. Psychiatric symptoms were rated by trained researchers using the Brief Psychiatric Rating Scale (BPRS), a 24-item scale measures the presence and severity of psychiatric symptoms. Each symptom is rated from 1 (‘not present’) to 7 (‘extremely severe’) and a summed score calculated to measure symptom severity. The scale has been shown to be valid, reliable and sensitive to change over time [26]. The objective social outcomes index (SIX) contains items relating to employment status, accommodation, living situation and social contact. The SIX provides a summary score ranging from 0 (poorest social situation) to 6 (best social situation). We also recorded psychiatric hospitalisations in the last 3 months.

Recruitment, attendance, group size, session duration, topics of discussion were assessed as process measures and qualitative interviews were used to capture the experience of participants. Interviews were conducted at the end of the intervention period using an interview topic guide designed to explore reasons for poor attendance, willingness to participate, patient experiences, and the perceived effectiveness of multifamily groups.

### Statistical analysis

A detailed statistical analysis plan was finalised and signed off prior to data analysis. Between-group comparisons are summarised using the mean, standard deviation and range for continuous variables, and frequencies and percentages for categorical variables.

Generalised mixed linear models were used for continuous outcome variables (MANSA, BPRS) to compare mean scores between the intervention and control groups, with fixed effects for treatment and baseline outcomes. Objective social outcomes (SIX) were measured using a proportional odds model with treatment fitted as a fixed effect. Fisher’s exact test was used to compare psychiatric hospitalisations between the intervention and control groups. Cohen’s *d* was derived as a standardised measure of effect. We conducted regression analyses on available cases based on intention-to-treat principles.

Qualitative data were analysed following the guidelines of Miles and Huberman (1994) using NVivo qualitative analysis software. Transcriptions were de-identified prior to analysis by removing all references to patients, clinicians or local services. All analyses were conducted in Bosnian and selected quotes were translated into English post-analysis.

## Results

### Participants

**Fig. 1:**
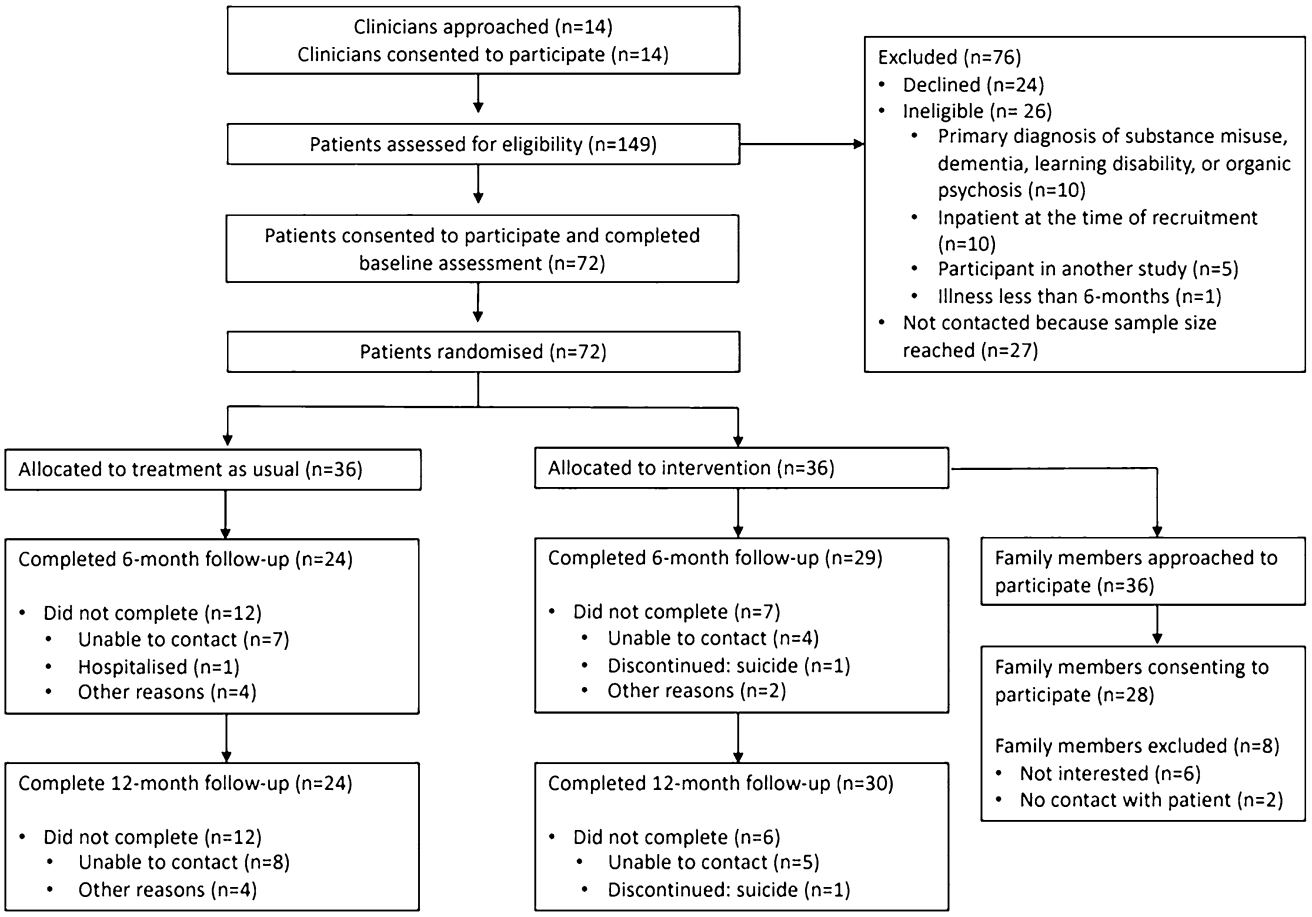
CONSORT diagram

We approached 14 clinicians (seven psychiatrists, three psychologists, two nurses and one social worker), all of whom agreed to participate in the trial. From the 149 outpatient records they screened for eligibility, 89 patients met with the research team and 72 provided informed consent. These 72 patients completed baseline assessment and were randomly allocated into either one of the six multifamily groups (*n* = 36) or the control arm (*n* = 36). The flow of patients through the study is detailed in the CONSORT diagram in Fig. 1.

The characteristics of the patients in the intervention and control group are shown in Table 1.

**Table 1:** Sample characteristics at baseline, *n* (%)

	Intervention group (*n* = 36)	Control group (*n* = 36)
Mean age in years (range)	45 (range: 26–69)	43 (range: 20–72)
Sex		
Male	12 (33%)	13 (36%)
Female	24 (67%)	23 (64%)
Marital status		
Single/unmarried	17 (48%)	17 (47%)
Married	15 (42%)	11 (31%)
Divorced	3 (8%)	5 (14%)
Widow/widower	1 (3%)	3 (8%)
Education		
Primary or less	4 (11%)	8 (22%)
Secondary	22 (61%)	22 (61%)
Tertiary or higher	10 (28%)	6 (17%)
Living with		
Family/partner	34 (95%)	32 (89%)
Alone	2 (6%)	4 (11%)
Employment		
Full time	8 (22%)	8 (22%)
Part time	1 (3%)	0 (0.0%)
Unemployed	13 (37%)	15 (42%)
Student	2 (6%)	3 (8%)
Invalid pension	6 (17%)	6 (17%)
Housewife/husband	1 (3%)	2 (6%)
Family pension	4 (11%)	3 (8%)

The two groups had similar socio-demographic and clinical characteristics. Overall, the mean age of patients recruited to the intervention was 45 years, and 67% were female. One-quarter were in full- or part-time employment, 42% were married and 95% lived with their family or partner.

Of the 36 family members nominated to participate in the intervention, 28 (78%) agreed to attend sessions. The mean age of participating family members was 53 years (range 23–71) and their most common relationship to the patient was parent (54%) followed by spouse (29%). Male and female family members were approximately equally represented (54% male).

### Intervention

Over the 6-month intervention period, the groups met on average five times (range 4–6). The mean number of sessions attended was 3.5 per patient (range 0–6) and 2.6 per family member (range 0–6). Patient attendance was 72% at the first session and 56% by the final session. The mean duration of each session was 95 min (range 60–110) and the mean number of attendees at each session (patients, family members and clinicians) was 7.6. The most frequent agreed topics for discussion were acceptance and relationships with family; coping with illness and available services; medication management and side effects; future plans; strategies for independent living; and benefits of leisure activities. Topics which were covered in only single meetings included the role of religion; disclosure of illness; societal attitudes to mental illness; impact of weather on mood disorders; and dealing with personal and family shame.

### Outcomes

Findings on the primary outcome and secondary outcomes at 6 and 12 months are shown in Table 2.

**Table 2:** Primary and secondary outcomes at baseline, 6 months and 12 months

Outcome	Intervention (*n* = 36)	Control (*n* = 36)	Test statistic	*p* value	Cohen’s *d*
Baseline					
MANSA (mean score, SD)	4.2 (0.8)	4.1 (0.6)			
SIX (mean score, SD)	4.1 (1.1)	3.9 (1.3)			
BPRS (mean score, SD)	32.3 (5.9)	33.8 (6.4)			
Psychiatric hospitalisation (count, %)	14 (38.9%)	23 (63.9%)			

Outcome	Intervention (*n* = 29)	Control (*n* = 24)	Test statistic	*p* value	Cohen’s *d*
6 months					
MANSA (mean score, SD)	5.2 (1.3)	4.3 (1.0)	*F* = 6.367	0.016	0.78
SIX (mean score, SD)	4.5 (1.2)	4.1 (1.3)	Proportional OR: 0.976	0.105	0.31
BPRS (mean score, SD)	31.6 (5.0)	31.8 (5.3)	*F* = 0.093	0.763	0.04
Psychiatric hospitalisation (count, %)	2 (6.9%)	8 (33.3%)	Fisher’s exact test	0.031	NA

Outcome	Intervention (*n* = 30)	Control (*n* = 24)	Test statistic	*p* value	Cohen’s *d*
12 months					
MANSA (mean score, SD)	4.7 (1.6)	3.3 (0.9)	*F* = 17.922	< 0.001	1.08
SIX (mean score, SD)	4.2 (1.4)	3.9 (1.0)	Proportional OR: 0.812	0.136	0.25
BPRS (mean score, SD)	30.5 (3.9)	35.0 (7.7)	*F* = 6.919	0.012	0.74
Psychiatric hospitalisation (count, %)	2 (6.7%)	3 (12.5%)	Fisher’s exact test	0.646	NA

*NA* not applicable, *OR* odds ratio

The primary outcome of subjective quality of life at 6 months showed a significant difference between the intervention and control group, with an effect size of 0.78. For secondary outcomes at 6-months, there were also significant differences in the proportion of participants experiencing a psychiatric hospitalisation between the intervention group (*n* = 2, 7%) and the control group (*n* = 8, 33%, *p* = 0.031), but no statistically significant difference in objective social outcomes or psychiatric symptoms.

At 12-month assessment, subjective quality of life was again more favourable in the intervention group. The effect size was 1.08. The intervention group also had significantly lower symptom levels with an effect size of 0.74. There were no statistically significant differences in psychiatric hospitalisations or objective social outcome scores between the two groups at 12 months.

### Qualitative interviews

Individual interviews were conducted with a convenience sample of 15 patients at the conclusion of the intervention period. Attendance at multifamily group meetings was a frequently discussed topic in our interviews. While meetings were held at agreed times monthly, for some, family obligations prevented regular attendance at group meetings.

‘Well, for me personally, to start first of all, it is difficult because I am obliged to my children and the school, the home obligations that I have’ (Patient 067)

Others described the possibility that poor attendance by some family members reflected a fear of clinical mental health services and many patients stated that they would have preferred if meetings had not taken place in a hospital.

‘Well, I don't know, maybe they're actually afraid of both the psychiatrist and the psychologist. Most people immediately think of something ugly when you mention a psychologist and a psychiatrist. And I don't think most of them wanted to come because of that’ (Patient 038)

“It was an unusual place for me, I didn't mind being in the hospital, but maybe it would have been more natural if we were in a different environment, but I didn't mind’ (Patient 015)

In general, the intervention lived up to the expectations of participants at enrolment. Patients felt the meetings were informative and they valued the advice they received. Through shared experiences and socialising with others with their diagnosis, patients learnt coping strategies and solutions for their everyday challenges.

‘I listened to a lot of other people, how they are and what they went through in life and so on. That's a nice social setting, story and that's all I expected and imagined and assumed that it would be like before it started’ (Patient 033)

Other patients described perceptions of improved quality of life since attending multifamily session and the impact the intervention had on their family relationships and the level of support they received.

‘I think that group therapy is the best possible therapy, if we all talk about our problems and lives together. And you can compare your life with other people's lives. I have a feeling that my quality of life has improved, that I look at some things differently.’ (Patient 067)

‘Mom is even more caring now, somehow she worries more, but she saw that we are not the only ones, that there are other people with similar diagnoses. They didn't pay that much attention before ... And now they keep asking me if I can do it alone, if I need help.’ (Patient 004)

‘My relationship with my husband is now somehow very nice… so I think I have learned some lessons and benefits from it all.’ (Patient 067)

## Discussion

This is the first randomised controlled trial of brief multifamily groups for patients with schizophrenia in a low- and middle-income country. The intervention appears feasible and acceptable in this context. Over 80% of patients approached agreed to participate in the intervention, sessions were well-attended, and in qualitative interviews patients described the value of mutual learning through shared experiences and the strengthening of family relationships during the intervention. Most importantly, the multifamily groups—despite being provided only six times—led to statistically and clinically significant improvements in the primary outcome, quality of life at 6 months, and these improvements were maintained at 12 months. The effect sizes at both time points suggest a large effect. We also found significant improvements in psychiatric hospitalisations at 6 months and psychiatric symptoms at 12 months.

### Strengths and limitations

The trial design was well implemented with good adherence rates in the intervention group, reasonable follow-up rates in both groups and blinded assessments. Outcomes were assessed on validated and widely used instruments, and quantitative measures were complemented by qualitative interviews. The statistical analysis was pre-specified and yielded a clear result with a large effect size on the primary outcome.

However, the study also has limitations. While sessions were well attended by patients (mean 3.5 sessions), attendance was poorer for family members (mean 2.6 sessions), and eight patients did not have a family member agree to participate. The loss of patients at follow-ups (26% at 6-months, 24% at 12 months) is comparable to other studies of psychosocial interventions for patients with schizophrenia [27], but it may still have introduced bias. A further limitation is that the 12-month follow-up for some patients was done when the COVID-19 pandemic had already started and restrictions had been introduced in Bosnia and Herzegovina to control the spread of the virus. The pandemic and the restrictions may have led to the deterioration of the objective social situation, which was seen in patients in both arms between the six and the 12-month follow-ups, and possibly also impacted on patients’ subjective quality of life during that period. Whilst pandemic and restrictions affected patients in both groups, they may have increased or decreased the effect of the intervention. However, this does not change the main result of the trial since the primary outcome was quality of life at 6 months which was assessed at a time before any restrictions had been imposed.

### Interpretation and implications

This is the first randomised controlled trial on any family intervention in Bosnia and Herzegovina and there is limited evidence on such interventions from the Southeast-Europe region [28]. Our results align with the wider literature from other low- and middle-income countries showing evidence of improved mental health outcomes for people with severe mental illness attending family involvement interventions [12, 29–31]. The effect size on the primary outcome in our trial was large—larger than in most other trials on psycho-social interventions in patients with schizophrenia—and maintained at the 12-month follow-up when there had been no group meetings for 6 months. Given the large effect size, one can have different views about whether the findings justify wide implementation right away or whether further larger trials should be conducted first. Considering the low costs and low risk of the intervention, its substantial and statistically significant effect in this trial, and the consistency with other findings in the literature, one might argue that delaying the implementation by several years to wait for the results of another trial focusing solely on effectiveness may not be justified. Instead, implementation trials—potentially as hybrid trials also assessing effectiveness—may test strategies for implementing multifamily groups in routine care within the healthcare systems of Bosnia and Herzegovina and other countries in the Southeast-European region [32].

One can only speculate about the reason for why such a brief and low-cost intervention of up to six meetings can have such a large and sustained effect. Families may be particularly cohesive, connected and supportive in the culture in Bosnia and Herzegovina, as possibly in many other low- and middle-income countries. This may have facilitated the mobilisation of existing resources and strengths in the families [10]. Although only one or two family members were involved in the meetings, it may have enabled patients to tap into and benefit from extensive support in wider family networks. Also, the mutual respect and appreciation conveyed in these groups may have raised the self-esteem and confidence of patients and helped them to a more active societal role which in turn may improve their quality of life. The findings may be related to the results of two other recent randomised-controlled trials conducted in Bosnia and Herzegovina. In these trials, two further low-cost resource-oriented interventions for patients with severe mental illnesses were tested, i.e. befriending through volunteers [33] and improving the patient-clinician meetings using the DIALOG+ approach [34]. The interventions also showed substantial benefits. Wider future research should explore the reasons for the large effect sizes of all these resource-oriented interventions and identify common and specific processes in more detail which might help to modify the interventions and make them even more effective.

Independently of the precise processes that explain the effect, the findings suggest that this low-cost intervention can lead to substantial improvements. In most settings, multi-family groups with only six meetings should be relatively straightforward to arrange. Expenses of services are required only for clinician time for six meetings and the space, and the costs for these are limited. Thus, there is a potential for the scaling up of multifamily groups in Bosnia and Herzegovina and indeed other low- and middle-income countries. This could involve both vertical scaling-up (i.e. the institutionalisation of this intervention within the national healthcare system)[35] or the expansion of delivery into other settings, such as inpatient care, where increasing evidence points to increasing family involvement being both feasible and acceptable, and providing increased satisfaction with treatment [13].

## Conclusion

Multifamily groups are a low-cost intervention that is feasible, acceptable and effective in patients with schizophrenia in Bosnia and Herzegovina. It may not be an appropriate approach for all patients with schizophrenia, but those patients who accept the intervention and participate tend to improve their quality of life substantially. Whilst further research should explore the processes, the findings suggest that brief multifamily groups should be widely provided for patients with schizophrenia, particularly in settings with limited resources where more specialised family interventions are not routinely available.
