# Correction to: Multifamily groups for patients with schizophrenia: an exploratory randomised controlled trial in Bosnia and Herzegovina

**DOI:** 10.1007/s00127-022-02288-w

**Published:** 2022-04-11

**Authors:** M. Muhić, S. Janković, H. Sikira, S. Slatina Murga, M. McGrath, C. Fung, S. Priebe, A. Džubur Kulenović

**Affiliations:** 1 Department of Psychiatry, University of Sarajevo, Sarajevo, Bosnia and Herzegovina; 2 Faculty of Medical Sciences, University of Kragujevac, Kragujevac, Serbia; 3 Unit for Social and Community Psychiatry, WHO Collaborating Centre for Mental Health Services Development, Queen Mary University of London, London, UK

## Correction to: Social Psychiatry and Psychiatric Epidemiology 10.1007/s00127-022-02227-9

In the original publication of the article, the initial of Professor Stefan Priebe has been incorrectly published as ‘P’ instead of’S’. The correct author name is given in this erratum.

The original article was corrected.

